# A convolutional neural network for total tumor segmentation in [^64^Cu]Cu-DOTATATE PET/CT of patients with neuroendocrine neoplasms

**DOI:** 10.1186/s13550-022-00901-2

**Published:** 2022-05-28

**Authors:** Esben Andreas Carlsen, Kristian Lindholm, Amalie Hindsholm, Mathias Gæde, Claes Nøhr Ladefoged, Mathias Loft, Camilla Bardram Johnbeck, Seppo Wang Langer, Peter Oturai, Ulrich Knigge, Andreas Kjaer, Flemming Littrup Andersen

**Affiliations:** 1 Department of Clinical Physiology and Nuclear Medicine & Cluster for Molecular Imaging, Copenhagen University Hospital – Rigshospitalet & Department of Biomedical Sciences, University of Copenhagen, Copenhagen, Denmark; 2 ENETS Neuroendocrine Tumor Center of Excellence, Copenhagen University Hospital – Rigshospitalet, Copenhagen, Denmark; 3 Department of Oncology, Copenhagen University Hospital – Rigshospitalet, Copenhagen, Denmark; 4 Department of Clinical Medicine, University of Copenhagen, Copenhagen, Denmark; 5 Department of Clinical Endocrinology and Surgical Gastroenterology, Copenhagen University Hospital – Rigshospitalet, Copenhagen, Denmark

**Keywords:** Tumor segmentation, Artificial intelligence, Neuroendocrine neoplasms, [^64^Cu]Cu-DOTATATE PET, Prognostication

## Abstract

**Background:**

Segmentation of neuroendocrine neoplasms (NENs) in [^64^Cu]Cu-DOTATATE positron emission tomography makes it possible to extract quantitative measures useable for prognostication of patients. However, manual tumor segmentation is cumbersome and time-consuming. Therefore, we aimed to implement and test an artificial intelligence (AI) network for tumor segmentation. Patients with gastroenteropancreatic or lung NEN with [^64^Cu]Cu-DOTATATE PET/CT performed were included in our training (*n* = 117) and test cohort (*n* = 41). Further, 10 patients with no signs of NEN were included as negative controls. Ground truth segmentations were obtained by a standardized semiautomatic method for tumor segmentation by a physician. The nnU-Net framework was used to set up a deep learning U-net architecture. Dice score, sensitivity and precision were used for selection of the final model. AI segmentations were implemented in a clinical imaging viewer where a physician evaluated performance and performed manual adjustments.

**Results:**

Cross-validation training was used to generate models and an ensemble model. The ensemble model performed best overall with a lesion-wise dice of 0.850 and pixel-wise dice, precision and sensitivity of 0.801, 0.786 and 0.872, respectively. Performance of the ensemble model was acceptable with some degree of manual adjustment in 35/41 (85%) patients. Final tumor segmentation could be obtained from the AI model with manual adjustments in 5 min versus 17 min for ground truth method, *p* < 0.01.

**Conclusion:**

We implemented and validated an AI model that achieved a high similarity with ground truth segmentation and resulted in faster tumor segmentation. With AI, total tumor segmentation may become feasible in the clinical routine.

**Supplementary Information:**

The online version contains supplementary material available at 10.1186/s13550-022-00901-2.

## Background

Neuroendocrine neoplasms (NENs) originate from neuroendocrine cells with the primary tumor most often located in the lungs [1], gastrointestinal tract or pancreas [2]. The clinical course for patients diagnosed with NEN ranges from indolent to highly aggressive. NENs are often slow-growing and due to vague symptoms tumors are often diagnosed at a late stage with metastatic disease. Most NENs have overexpression of somatostatin receptors [3], which can be used for tumor imaging. Positron emission tomography (PET) for somatostatin receptor imaging (SRI) combined with anatomical imaging, e.g., computer tomography (CT), is essential for diagnosing, staging, treatment selection and follow-up of patients with NEN [4].

Previously, the use of maximal standardized uptake value (SUV_max_) in SRI PET of patients with NEN has been shown to be prognostic for progression-free survival, but not overall survival [5–8]. The degree of somatostatin receptor expression determined by SUV is linked with tumor differentiation, i.e., less somatostatin receptors in the dedifferentiated more aggressive tumors. Thus, the lesion with the lowest tracer uptake, rather than SUV_max_, would therefore be expected to be better linked with prognosis. Indeed, we recently reported that by use of minimum lesion SUV_mean_ and total tumor volume, the prognostic value of SRI with [^64^Cu]Cu-DOTATATE PET in patients with NEN could be greatly increased [9]. However, to obtain total tumor volume and minimum SUV_mean_, total tumor segmentation is required. Due to the often widely metastatic disease in patients with NEN this is time-consuming with a manual approach, thus hindering clinical implementation. We, therefore, introduced a standardized semiautomatic method for total tumor segmentation that could be performed with a median time of approximately 20 min depending on tumor burden. However, to increase the likelihood of clinical implementation of these prognostic measures, we believe further automatization and faster tumor segmentation is needed. We, therefore, set out to employ a deep-learning approach for total tumor segmentation in [^64^Cu]Cu-DOTATATE PET of patients with NEN. One widespread method for image segmentation is using the U-net structure for the artificial intelligence (AI) network [10], which has been used for, for example, segmentation of bone metastasis in breast cancer patients [11], of cervical tumors [12] and of gliomas [13] in PET/CT. To the best of our knowledge, this has, however, not been applied to tumor segmentation in patients with NEN. Adapting a network of the U-Net architecture could therefore be a promising step in further automatization of tumor segmentation of NENs.

Hence, we aimed to implement and evaluate an AI model for tumor segmentation of NENs and determine if the performance of the model could prove useful for assisting or replacing our previously proposed standardized semiautomatic method for total tumor segmentation. Further, the model was implemented into clinically used software.

## Methods

### Patients

#### Dataset 1

From 2009 to 2013, we retrospectively included 127 patients available with histopathologically confirmed NENs that underwent [^64^Cu]Cu-DOTATATE PET /CT performed 1 h after injection of approximately 200 MBq [^64^Cu]Cu-DOTATATE PET. All images were acquired with a Siemens Biograph 40 or 64 TruePoint PET/CT and reconstructed with TrueX algorithm (Siemens Medical Solution). Settings were as follows; 3 iterations, 21 subsets, 2 mm Gaussian post-filter smoothing, 336 × 336 matrices of 2 × 2 × 3 mm^3^ voxels. CT-based attenuation correction was applied. A diagnostic quality CT scan with iodine intravenous contrast was performed before the PET. To ensure quantitatively accurate measurements between the different PET/CT scanners, we perform a quality control every 2 weeks, testing they are calibrated to measure within our acceptance range (5%). We excluded patients with no signs of NEN due to previous radical surgery. For patients included; age, gender, site of primary tumor, Ki67 index and WHO grade (Grade 1 (Ki67 < 3%), Grade 2 (Ki67 3–20%) and Grade 3 (Ki67 > 20%) were recorded.

#### Dataset 2

From 2018 to 2019, we retrospectively included 31 patients with histopathologically confirmed NEN that underwent [^64^Cu]Cu-DOTATATE PET /CT. All PET/CT were acquired with a Siemens Biograph 128 mCT using the same setup and reconstruction routine as described above.

#### Dataset 3

Also from 2018 to 2019, we retrospectively included 10 patients with known NEN referred for [^64^Cu]Cu-DOTATATE PET/CT but found to have no signs of NEN on PET/CT due to radical treatment and therefore serves as negative controls in this study. Images were acquired as described for dataset 2.

To ensure quantitatively accurate measurements between the different PET/CT scanners, we perform a quality control every 2 weeks, testing they are calibrated to measure within our acceptance range (5%). A Danish Patient Safety Authority approval was obtained (31-1521-453), and obtained informed consent was waived for the included patients.

### Ground truth label methodology

Ground truth labels were created using a previously described standardized procedure for segmentation of NENs in [^64^Cu]Cu-DOTATATE PET /CT images [9]. In brief, a region of interest (ROI) is drawn within the normal liver on the PET image. The SUV_mean_ value of this ROI is then used to calculate a threshold value for the rest of the image. The threshold value is given as

$$Threshold = \left( {1.5 \cdot SUV_{mean} } \right) + \left( {2 \cdot SD} \right)$$

with SD being the standard deviation within the ROI. In case of complete metastatic liver involvement the ROI is drawn in the spleen. The threshold formula based on normal spleen uptake has been adapted to the higher physiological uptake in the spleen and is given as

$$Threshold = \left( {0.67 \cdot SUV_{mean} } \right) + \left( {2 \cdot SD} \right)$$

Consequently, every voxel from the PET image with a value larger than the threshold is by default segmented. To reduce segmentation due to noise, all volumes < 0.1 mL (< 9 voxels) are automatically removed. Finally, a physician (E.A.C) manually corrected the segmentation by removing volumes with high physiological uptake (typically bladder, adrenal glands and kidney) and false-positive segmentations yielding the ground truth label.

### Convolutional neural network

**Fig. 1:**
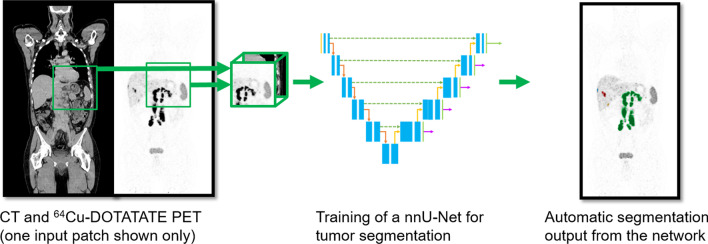
Total tumor segmentation of neuroendocrine neoplasms by a convolutional neural network

We applied the U-net architecture which involves a de-convolutional path for extraction of features in the images and an up-convolutional path for localization of the features (Fig. 1). The well-established nnU-Net framework was used for automatically setting up the U-net including preprocessing, training, inference and post-processing of data [14]. We used default nnU-Net settings for training the network with PET and CT images as input, and our ground truth label as target. Details regarding the preprocessing, network architecture and hyperparameters are shown in Supplementary Table 1.

### Training and validation of the network

**Fig. 2:**
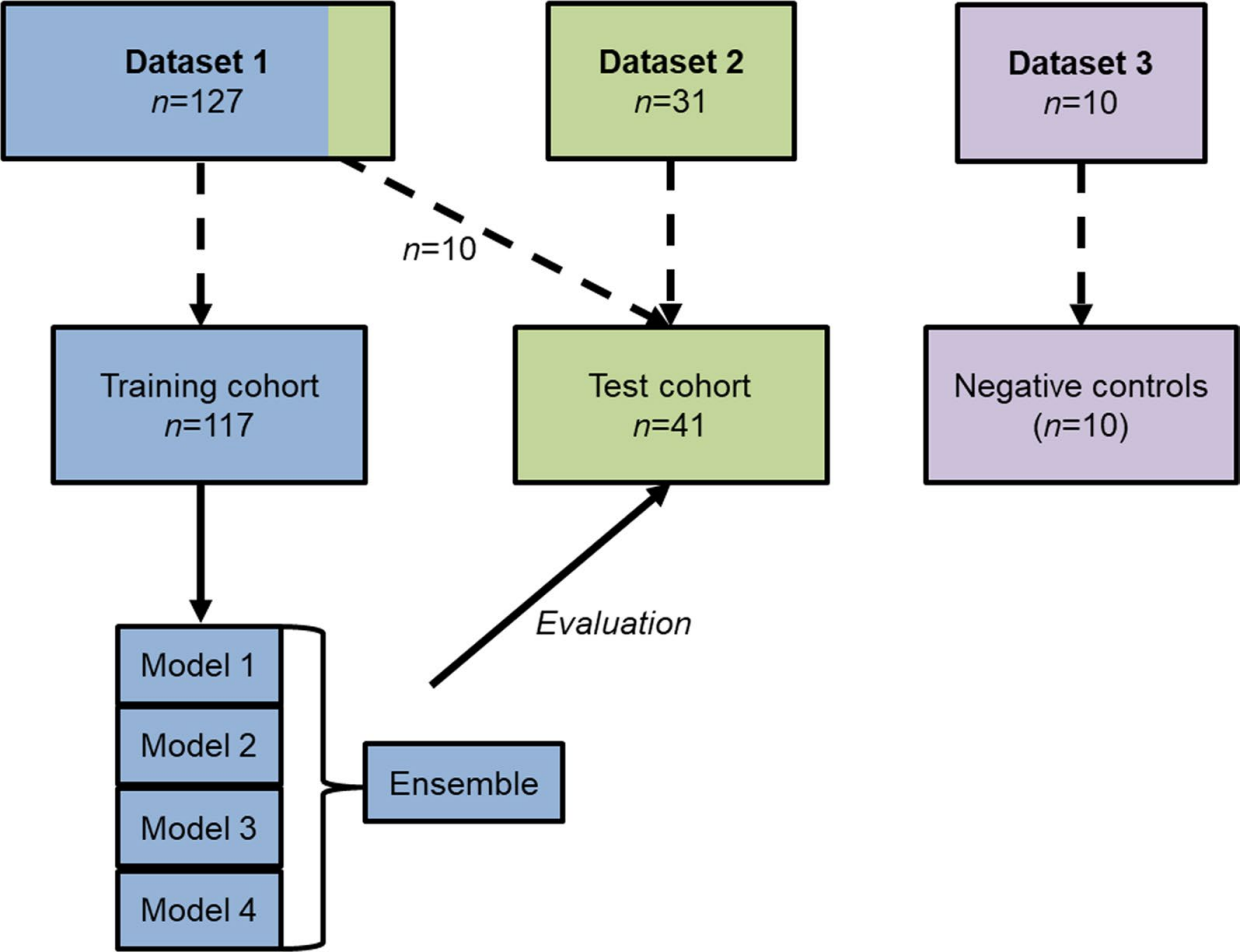
Illustration of data used for training and test. The models and the ensemble hereof were inferred on the test cohort. Boxes are not drawn to scale

For training and validation, a randomly selected subset of 117 [^64^Cu]Cu-DOTATATE PET/CTs from dataset 1 were used (Fig. 2). Cross-fold training was performed in accordance with the nnU-Net procedure. The training results in a model from each fold, where individual pixels are predicted to be either segmented or not with some probability by each model. An ensemble of the models was created by averaging these probabilities before each pixel was assigned to be segmented or not in the ensemble model. Post-processing with removal of any segmentation outside the patient’s body was performed using the PET signal to automatically outline the body contour. Furthermore, segmentations < 0.1 mL (< 9 voxels) were automatically removed.

### Testing of models

The remaining 10 patients from dataset 1 (not used in training) and 31 patients from dataset 2 constituted the test cohort. Patients from both dataset 1 and 2 were used for testing to control for possible effect of different PET/CT systems used. The automatically post-processed models were evaluated by the following metrics in the test cohort.

$$Sensitivy = \frac{TP}{{TP + FN}}$$

$$Precision = \frac{TP}{{TP + FP}}$$

$$Dice = \frac{2 \cdot TP}{{2 \cdot TP + FP + FN}}$$

where TP, FP, TN and FN denote true/false positive/negative. These metrics were used to select which model should be further evaluated. The selected output were reformatted into DICOM RT structures and imported into Mirada DBx 1.2.0 software package (Mirada Medical Ltd., Oxford, UK). An arbitrary scoring scheme (Table 1) focusing on number of false positive and false negative lesions was applied to evaluate the automatically post-processed AI segmentation in the test cohort (*n* = 41). Time spent on any necessary manual correction was recorded in a randomly selected subgroup (*n* = 10), blinded to, and compared with time spent on ground truth labeling. Scoring of the AI segmentations in the test cohort was performed by a physician (E.A.C). Additionally, the AI model was tested on patients from dataset 3, i.e., patients without any signs of NEN on PET/CT due to radical surgery. These patients were judged by the number of segmentations (if any) and volume of the segmentation.

**Table 1:** Evaluation scheme of AI segmentations

Rating	Criteria
1. Perfect/optimal	The segmentation is as good as manual segmentation, that is, no false positive or false negative segmentations
2. Optimal with minor adjustments	The segmentation contains all lesions and only minor* false positives or false negatives
3. Acceptable with minor adjustments	The segmentation contains the majority of the lesions (at least 1 and ≤ 3 missing) and ≤ 2 false positive segmentations. Additionally only minor* false positives or false negatives
4. Acceptable with major adjustments	The segmentation contains most of the lesions (at least 1 and ≤ 6 missing) and ≤ 4 false positive segmentations. Additionally only minor* false positives or false negatives
5. Non-usable	The segmentation does not contain enough of the lesions (≥ 7 lesions missing or no lesions segmented if less than 7 lesions present) or too many false positives (≥ 5) for correction to be meaningful

* Minor is defined as only parts of a predicted lesion are wrong

### Statistical analyses

Data are reported as mean and standard deviation unless otherwise indicated. To assess difference between groups we used chi-squared test for categorical data and unpaired and paired t-test for continuous data, as appropriate. A P-value < 0.05 was considered statistically significant. Statistical analyses were performed in R version 3.6.0 (R Foundation for Statistical Computing).

## Results

### Patients

The datasets for training and testing the models were similar in regards to age, gender and site of primary tumor. Patients most often had small intestinal primary tumors. Patients in the test cohort were more often classified as WHO Grade 2, but the Ki67 index did not differ significantly (Table 2). All but one patient in the test cohort had multiple lesions, with a median of 25 lesions (interquartile range: 41).

**Table 2:** Demographic data for patients with neuroendocrine neoplasms

Training and test datasets	Training	Test	Overall
	(*N* = 117)	(*N* = 41)	(*N* = 158)
Mean age, year	62 (SD, 11)	65 (SD, 10)	63 (SD, 10)
Gender			
Male	63 (54)	24 (59)	87 (55)
Female	54 (46)	17 (42)	71 (45)
Site of primary			
Small intestine	67 (57)	23 (56)	90 (57)
Pancreas	23 (20)	9 (22)	32 (20)
Lung	7 (6)	5 (12)	12 (8)
Cecum	6 (5)	2 (5)	8 (5)
Extrahepatic biliary tract	2 (2)	0 (0)	2 (1)
Esophagus	1 (1)	0 (0)	1 (1)
Gastric	1 (1)	0 (0)	1 (1)
Unknown primary NEN	10 (9)	2 (5)	12 (8)
WHO*			
Grade 1	29 (25)	3 (7)	32 (20)
Grade 2	75 (64)	32 (78)	107 (68)
Grade 3	6 (5)	6 (15)	12 (8)
Missing	7 (6)	0 (0)	7 (4)
Mean Ki67, %	8 (SD, 14)	11 (SD, 8)	9 (SD, 12)

Negative controls	Training	Test	Overall
	(*N* = 0)	(*N* = 10)	(*N* = 10)
No disease	0 (0)	10 (100)	10 (100)

^*^Denotes statistically significant difference in distribution of WHO grades between training and test data (*p* = 0.013). Data are number followed by percentage in parentheses, unless otherwise indicated. Percentage were rounded and may not add up to 100%

### Output from nnU-Net

The models, as well as the ensemble of the models, were evaluated using the defined metrics in the test cohort (*n* = 41). Since no model performed best in regards to both pixel and lesion-wise metrics (Table 3), the ensemble was chosen for evaluation of FP/FN lesions. Because the ensemble is a combination of the different models, it comprises more variation in the dataset and should be the more robust model/choice for new cases.

**Table 3:** Metrics for AI segmentations without manual adjustments applied to the test cohort (*n* = 41)

Pixel-wise	AI model				
	Model 1	Model 2	Model 3	Model 4	Ensemble
Dice	0.801 (0.206)	**0.817** (0.176)	0.768 (0.234)	0.763 (0.233)	0.801 (0.196)
Precision	0.772 (0.258)	**0.816** (0.223)	0.752 (0.279)	0.787 (0.258)	0.786 (0.250)
Sensitivity	**0.893** (0.173)	0.860 (0.180)	0.869 (0.182)	0.821 (0.231)*	0.872 (0.177)

Lesion-wise					
Dice	0.847 (0.286)	0.828 (0.264)	0.809 (0.268)	0.803 (0.258)	**0.850** (0.278)
Sensitivity	**0.854** (0.230)	0.827 (0.234)	0.843 (0.228)	0.831 (0.243)	0.844 (0.238)

All values calculated as mean of the 41 patients of the test cohort with standard deviation in parentheses. Bold numbers mark the highest value across the models/ensemble in each evaluation metric. *Denotes statistically significant difference in sensitivity between Model 4 and Model 1 (*p* = 0.017)

### Evaluation of AI segmentations

**Fig. 3:**
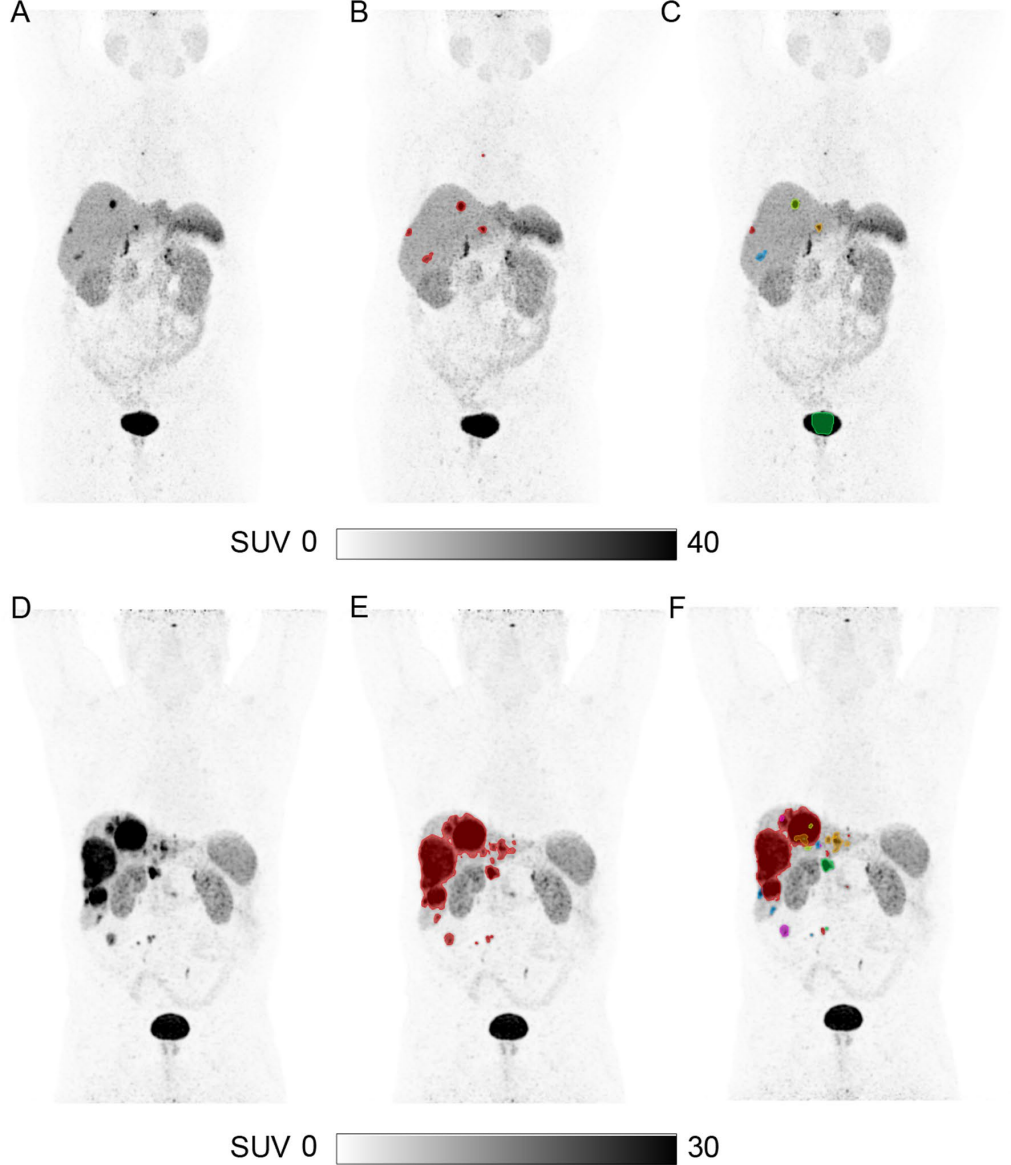
Representative examples of the segmentations from the AI model for two patients. Maximum intensity projection [^64^Cu]Cu-DOTATATE PET without tumor segmentation (**A**, **D**). Ground truth segmentation of tumor (**B**, **E**). AI predicted segmentations—no manual adjustments performed (**C**, **F**). In the AI output, all separate segmentations are given a unique color, e.g., red, blue, green, making manual adjustment with deletion of a segmentation easy and fast (e.g., part of the bladder was erroneously segmented in **C**)

**Fig. 4:**
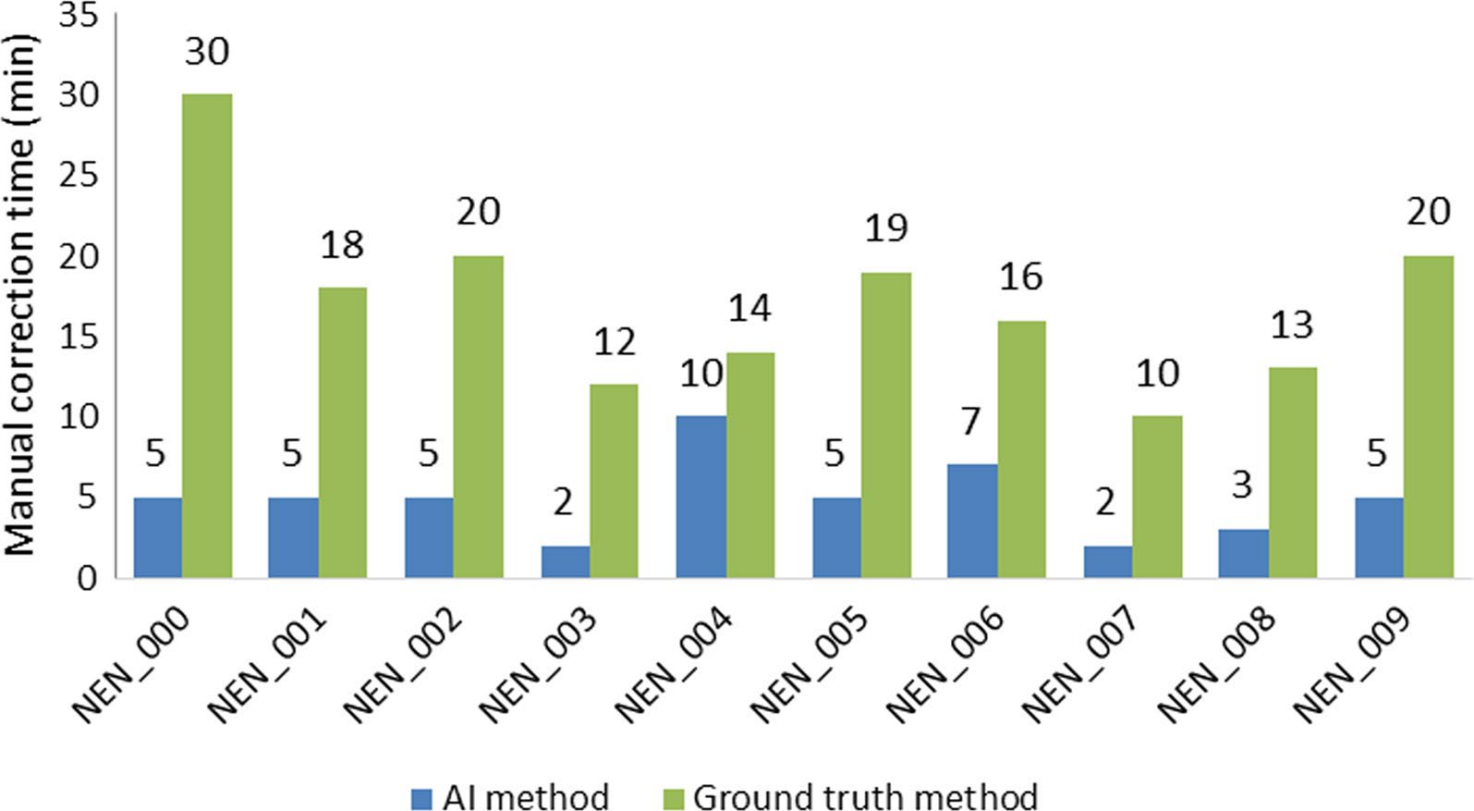
Boxplot depicting time spent on manual correction to obtain the final total tumor segmentation

All 41 patients in the test cohort were evaluated and scored according to the scoring scheme (Table 1) with the detailed description reported in Supplementary Table 2. Examples of two patients are shown in Fig. 3. In 7/41 (17%) patients the AI segmentation required no manual adjustments to obtain the final total tumor segmentation (Table 4). In 35/41 (85%) of patients, the AI segmentation was considered acceptable with either no or minor/major adjustment before the final total tumor segmentation was obtained. The time spent on any manual correction was compared between the output from the AI segmentation and from the standardized semiautomatic method used as ground truth. In the subgroup of 10 randomly selected patients, less time was needed to correct the segmentation when using the segmentation from the AI model (median 5 min) versus the semiautomatic method (median 17 min), *p* < 0.01 (Fig. 4). Further, the ensemble model was applied to the negative controls and in 5/10 (50%) no segmentations were seen, and in 9/10 (90%) cases, the volume segmented was ≤ 1.5 mL (e.g., physiological uptake in adrenal gland). The median segmented volume was 0.15 mL. In one negative control patient, 56 volumes were segmented with a total volume of 68.6 mL with almost all of the segmentations placed in normal liver tissue. No explanation for the false positive segmentations could be identified when reevaluating that particular patient.

**Table 4:** Evaluation of number of false-positive/false-negative segmentations by AI without manual adjustments

Evaluation score*	*n* = 41
1 (Perfect/optimal)	7 (17%)
2 (Optimal with minor adjustments)	3 (1%)
3 (Acceptable with minor adjustments)	19 (46%)
4 (Acceptable with major adjustments)	6 (15%)
5 (Non-usable)	6 (15%)

*Defined in Table 1

## Discussion

Our most important finding was that time spent on the laborious task of total tumor segmentation in patients with NEN can be reduced from 20 to 5 min by applying a convolutional neural network. The majority of the automatically performed segmentations by the network were useable for obtaining total tumor segmentation with either no or only minor degree of manual adjustments. Hence, the method proposed in our study serves to further automate the process of total tumor segmentation. Recently, we and others have reported the prognostic power of total tumor volume determined in SRI PET [9, 15–19]. Furthermore, with total tumor segmentation, several metrics may be derived in the setting of multiple lesions, e.g., the lesion with the lowest SUV_mean_ can easily be determined. Underscoring the value of total tumor segmentation, we recently reported the prognostic value of a combined approach of total tumor volume and minimum SUV_mean_ [9]. The reduced time needed for performing total tumor segmentation presented in the current study may further improve the clinical adaptation and feasibility in the clinical routine.

In our train cohort, we trained the nnU-Net convolutional neural network. In the test cohort, our proposed AI model without manual adjustments had a pixel and lesion-wise dice score of 0.801 and 0.850, respectively. This is comparable with other proposed models for tumor segmentation [13, 20, 21]. More importantly, by implementing the segmentation in a clinical imaging viewer, the segmentations could be judged, adjusted and verified by a physician. Of note, by reformatting the output to DICOM RT structures, it could easily be integrated into other clinical imaging viewers. From the evaluation it was seen that most (46%) of the AI segmentations of the 41 patients were scored as “Acceptable with minor adjustments.” Only 15% were scored as non-usable due to the number of manual adjustments needed, leaving 85% of the segmentations as useable for obtaining total tumor segmentation. Also, in the negative control cases the model performed well by segmenting a volume of < 1.5 mL in 90% and no segmentations in 50% of the 10 cases. From both the test cohort and negative controls, however, it is evident that the proposed model cannot stand alone. Manual adjustments are necessary in some cases, and the AI segmentations should always be examined by a physician. Compared with the previously proposed methodology for a standardized semiautomatic segmentation, the AI model serves as an improved starting point for the physician, and may be corrected in 5 min as compared to the previously reported 20 min [9]. The AI predicted segmentations should be viewed as an aid for performing total tumor segmentation, which, however, ultimately is determined by the physician performing the adjudication. The AI predicted segmentations should not give rise to altered clinical staging of the patients as compared to a manual total tumor segmentation approach, unless true positive lesions were overlooked by a manual approach. The manual adjustments may involve removing false positive segmentations, e.g., the adrenal gland or bladder, and/or adding lesion(s) not included in the segmentation. With the unique label for each segmented volume entire false positive segmentations are easily removed. The manual addition of single lesions can be done using several approaches, e.g., as seed point, where a seed is placed in the lesion, and a SUV threshold is used for determining the outline of the lesion. The threshold may be based on the standardized method we previously proposed, where the patient specific threshold is a based on tracer uptake in the normal liver. Other methods include a fixed threshold or adaptive threshold (e.g., 40% SUV_max_).

As the model is derived from a training cohort of 117 patients, it is evident given the large variation in primary tumor site and sites of metastatic lesions that the model may be further improved by having larger training cohorts. By implementing the model for segmentation, new training cohorts may be more easily generated given the reduction in time spent on performing tumor segmentation. Ideally, the model should also be validated in an external cohort. [^64^Cu]Cu-DOTATATE was developed and clinically implemented at our department at Rigshospitalet, Denmark, but has not been available at other sites until the recent Food and Drug Administration approval [22]. It is therefore likely that the proposed model could be externally validated in the future. The trained AI model may not be directly applied to a setting with another PET tracer for SRI, e.g., ^68^ Ga-DOTATOC, due to variations in uptake patterns in both normal tissues and lesions [23]. Some issues should be addressed regarding the proposed AI model. For both ground truth segmentation and by post-processing of the model output, all segmentation < 0.1 mL were automatically removed. With a voxel size of approximately 2 × 3 × 3 mm^3^ = 0.012 mL, the deletion of all lesions below 0.1 mL corresponds to lesions below 9 voxels. This was done to reduce segmentation of noise. Furthermore, in the training and test cohorts small intestinal and pancreatic primary tumors were most frequent; hence, the AI model may perform best in such cases.

## Conclusion

We implemented and validated an AI method that achieved a high concordance with the ground truth label and resulted in much faster tumor segmentation. In the majority of patients, the AI segmentation was useable with either no or minor manual adjustments required. By applying this approach of total tumor segmentation, prognostication by quantitative measures of [^64^Cu]Cu-DOTATATE PET may become more feasible and implemented in the clinical routine.

## Supplementary Information

**Additional file 1.** **Supplementary Table 1**. Data pre-processing, network details and hyperparameters for nnU-Net extracted by nnUNet_plan_and_preprocessing. **Supplementary Table 2**. Full list of evaluation of all 41 patients of the test cohort. Patients with ID NEN_000 – NEN_009 were from dataset 1 and the remainder from dataset 2.

## Data Availability

The trained AI model can be shared upon reasonable request to the corresponding author.

## Abbreviations

NEN: Neuroendocrine neoplasm
SRI: Somatostatin receptor imaging
SUV: Standardized uptake value
PET: Positron emission tomography
CT: Computer tomography
AI: Artificial intelligence
ROI: Region of interest
SD: Standard deviation
TP: True positive
FP: False positive
TN: True negative
FN: False negative
