# Supplementary material for: A convolutional neural network for total tumor segmentation in [64Cu]Cu-DOTATATE PET/CT of patients with neuroendocrine neoplasms
Source: EJNMMI Res. 2022 May 28;12:30. doi: 10.1186/s13550-022-00901-2 (PMC9148347; doi:10.1186/s13550-022-00901-2)
Supplement: Supplementary file 1 — Additional file 1. Supplementary Table 1. Data pre-processing, network details and hyperparameters for nnU-Net extracted by nnUNet_plan_and_preprocessing. Supplementary Table 2. Full list of evaluation of all 41 patients of the test cohort. Patients with ID NEN_000 – NEN_009 were from dataset 1 and the remainder from dataset 2. [file 13550_2022_901_MOESM1_ESM.docx]

# A convolutional neural network for total tumor segmentation in [^64^Cu]Cu-DOTATATE PET/CT of patients with neuroendocrine neoplasms

Esben Andreas Carlsen^1, 2^ *, Kristian Lindholm^1, 2^ *, Amalie Hindsholm^1,2^, Mathias Gæde^1,2^, Claes Nøhr Ladefoged^1,2,^ Mathias Loft^1, 2^, Camilla B Johnbeck^1, 2^, Seppo W. Langer^2,3^, Peter Oturai ^1, 2^, Ulrich Knigge^2,4^, Andreas Kjaer^1,2,^ **^,†^, Flemming Andersen^1,2^ **

* Shared first author

** Shared senior author

^1^ Dept. of Clinical Physiology and Nuclear Medicine & Cluster for Molecular Imaging, Copenhagen University Hospital – Rigshospitalet & Dept. of Biomedical Sciences, University of Copenhagen, Copenhagen Denmark

^2^ ENETS Neuroendocrine Tumor Center of Excellence, Copenhagen University Hospital – Rigshospitalet, Copenhagen, Denmark

^3^ Dept. of Oncology, Dept. Clinical Medicine, Copenhagen University Hospital – Rigshospitalet and University of Copenhagen, Copenhagen, Denmark

^4^ Depts. of Clinical Endocrinology and Surgical Gastroenterology, Copenhagen University Hospital – Rigshospitalet, Copenhagen, Denmark

^†^ Correspondence to Prof. Andreas Kjaer, MD, PhD, DMSc, Dept. of Clinical Physiology and Nuclear Medicine & Cluster for Molecular Imaging, Copenhagen University Hospital – Rigshospitalet & Dept. of Biomedical Sciences, University of Copenhagen, Blegdamsvej 9, DK-2100 Copenhagen, Denmark. Orcid: <https://orcid.org/0000-0002-2706-5547>. e-mail: [akjaer@sund.ku.dk](mailto:akjaer@sund.ku.dk)

Shared first authors: Esben Andreas Carlsen, MD, Dept. of Clinical Physiology and Nuclear Medicine & Cluster for Molecular Imaging, Copenhagen University Hospital – Rigshospitalet & Dept. of Biomedical Sciences, University of Copenhagen, Blegdamsvej 9, DK-2100 Copenhagen, Denmark. Orcid: <https://orcid.org/0000-0002-5231-4197>. e-mail: [esben.a.carlsen@gmail.com](mailto:esben.a.carlsen@gmail.com)

Kristian Lindholm, BSc, Dept. of Clinical Physiology and Nuclear Medicine & Cluster for Molecular Imaging, Copenhagen University Hospital – Rigshospitalet & Dept. of Biomedical Sciences, University of Copenhagen, Blegdamsvej 9, DK-2100 Copenhagen, Denmark. Orcid: <https://orcid.org/0000-0002-6240-4233>. e-mail: kristianlindholm@outlook.dk

## Supplementary

**Supplementary Table 1.** Data pre-processing, network details and hyperparameters for nnU-Net extracted by nnUNet_plan_and_preprocessing.

| **Data preprocessing** | | |
| --- | --- | --- |
| Input voxel spacing | | 3.0x2.036x2.036 mm^3^ |
| Median shape after pre-processing | | 374x366x366 voxels |
| Number of transaxial slices (min-max) | | 324-624 slices |
| Patch size | | 128x128x128 voxels |
| Normalization schemes: | | |
|  | CT | CT |
|  | PET | nonCT |
| **Network details** | | |
| Network | | 3d_fullres |
| Trainer | | nnUNetTrainerV2 |
| Initial learning rate | | 0.1 |
| Number of base features | | 32 |
| Batch size | | 2 |
| Max epochs | | 1000 |
| Number of batches per epoch | | 250 |
| net_conv_kernel_sizes | | [[3,3,3], [3,3,3], [3,3,3], [3,3,3], [3,3,3], [3,3,3]] |
| net_num_pool_op_kernel_sizes | | [[2,2,2], [2,2,2], [2,2,2], [2,2,2], [2,2,2]] |
| net_pool_per_axis | | [5,5,5] |
| **Data augmentation settings** | | |
| do_elastic | | False |
| elastic_deform_alpha | | (0.0, 900.0) |
| elastic_deform_sigma | | (9.0, 13.0) |
| p_eldef | | 0.2 |
| do_scaling | | True |
| scale_range | | (0.7, 1.4) |
| independent_scale_factor_for_each_axis | | False |
| p_independent_scale_per_axis | | 1 |
| p_scale | | 0.2 |
| do_rotation | | True |
| rotation_x | | (-0.52, 0.52) |
| rotation_y | | (-0.52, 0.52) |
| rotation_z | | (-0.52, 0.52) |
| rotation_p_per_axis | | 1 |
| p_rot | | 0.2 |
| random_crop | | False |
| random_crop_dist_to_border | | None |
| do_gamma | | True |
| gamma_retain_stats | | True |
| gamma_range | | (0.7, 1.5) |
| p_gamma | | 0.3 |
| do_mirror | | True |
| mirror_axes | | (0, 1, 2) |
| dummy_2D | | False |
| mask_was_used_for_normalization | | OrderedDict([(0, False), (1, False)]) |
| border_mode_data | | constant |
| all_segmentation_labels | | None |
| move_last_seg_chanel_to_data | | False |
| cascade_do_cascade_augmentations | | False |
| cascade_random_binary_transform_p | | 0.4 |
| cascade_random_binary_transform_p_per_label | | 1 |
| cascade_random_binary_transform_size | | (1, 8) |
| cascade_remove_conn_comp_p | | 0.2 |
| cascade_remove_conn_comp_max_size_percent_threshold | | 0.15 |
| cascade_remove_conn_comp_fill_with_other_class_p | | 0.0 |
| do_additive_brightness | | False |
| additive_brightness_p_per_sample | | 0.15 |
| additive_brightness_p_per_channel | | 0.5 |
| additive_brightness_mu | | 0.0 |
| additive_brightness_sigma | | 0.1 |
| num_threads | | 12 |
| num_cached_per_thread | | 2 |
| patch_size_for_spatialtransform | | [128, 128, 128] |

**Supplementary Table 2.** Full list of evaluation of all 41 patients of the test cohort. Patients with ID NEN_000 – NEN_009 were from dataset 1 and the remainder from dataset 2.

| Patient ID | Lesions (n) (ground truth) | Rating | False negative | False positive | Correction (minutes) |
| --- | --- | --- | --- | --- | --- |
| NEN_000 | 46 | 3 | 3 (lymph nodes) | Large liver lesions (too much segmented as lesion) | 5 |
| NEN_001 | 25 | 2 | Part of liver lesion false negative | None | 5 |
| NEN_002 | 49 | 3 | None | 1 (pancreas) | 5 |
| NEN_003 | 3 | 1 | None | None | 2 |
| NEN_004 | 63 | 3 | 3 (2 bone and 1 abdominal carcinomatosis plaque). 1 minor (part of lesion missing) | 2 (intestines) | 10 |
| NEN_005 | 6 | 3 | 1 (bone) | None | 5 |
| NEN_006 | 268 | 2 | Part of multiple liver lesions false negative. Low and heterogeneous tracer uptake in liver lesions. | None | 7 |
| NEN_007 | 7 | 1 | None | None | 2 |
| NEN_008 | 3 | 3 | None | 1 (liver) | 3 |
| NEN_009 | 12 | 4 | 4 (3 small lymph nodes and 1 small bone lesion) | None | 5 |
| NEN_226 | 14 | 3 | 1 (small liver lesion) | None | 6 |
| NEN_229 | 5 | 3 | 1 (minor) inguinal lymph node | 2 (small intestinal foci) | 3 |
| NEN_230 | 35 | 3 | 2 (small liver lesion and a lymph node | None | 10 |
| NEN_232 | 1 | 5 | No lesions segmented (low tracer uptake in tumor) | None | - |
| NEN_233 | 68 | 3 | 2 (abdominal carcinomatosis) | 1 (scrotum/contamination) | 8 |
| NEN_235 | 32 | 1 | None | None | 1 |
| NEN_236 | 25 | 5 | 11 (small bone lesions) | 2 (parotid gland and bone foci) | - |
| NEN_239 | 82 | 3 | 3 (liver lesions) | 1 (adrenal gland) | 6 |
| NEN_240 | 60 | 3 | None | 1 (adrenal gland) | 3 |
| NEN_241 | 32 | 4 | 6 (3 liver lesions and 3 lymph nodes) | None | 7 |
| NEN_243 | 26 | 4 | 2 (bone lesion and lymph node) | 3 (spleen, adrenal gland and ventricle (stomach) | 10 |
| NEN_244 | 118 | 5 | >20 bone lesions  Part of major cystic liver lesion not segmented | 2 (right kidney and spleen) | - |
| NEN_246 | 2 | 3 | Low tracer uptake. | 1 (adrenal gland) | - |
| NEN_249 | 48 | 5 | ~10 bone lesions | 1 (adrenal gland) | - |
| NEN_251 | 9 | 2 | None | 1 (minor) (urinary bladder) | 3 |
| NEN_252 | 41 | 1 | None | None | 2 |
| NEN_257 | 104 | 5 | >10 bone lesions missing | None | - |
| NEN_259 | 22 | 1 | None | None | 3 |
| NEN_260 | 57 | 3 | None | 2 (site of injection and spleen) | - |
| NEN_261 | 39 | 3 | 1 + part of lesion | None | 5 |
| NEN_263 | 30 | 3 | None | 1 (ventricle (stomach) | 2 |
| NEN_264 | 9 | 3 | None | 1 (ventricle (stomach) | 2 |
| NEN_265 | 67 | 5 | >10 bone lesions | 2 (adrenals glands) | - |
| NEN_270 | 6 | 4 | 4 bone lesions | None | 5 |
| NEN_271 | 3 | 1 | None | None | 2 |
| NEN_272 | 11 | 3 | 2 liver lesions | None | 8 |
| NEN_273 | 4 | 4 | 4 (small liver lesions) | 2 (bladder and part of adrenal gland) | 5 |
| NEN_275 | 12 | 1 | None | None | 3 |
| NEN_276 | 5 | 3 | 1 (lymph node) | 1 (bladder) | 3 |
| NEN_278 | 24 | 4 | None | 4 (adrenal gland, spleen, intestine, kidney) | 3 |
| NEN_280 | 39 | 3 | 2 (lymph nodes) + 2 (minor) (intestinal tumor + lymph node) | None | 5 |
